# Central amygdala neuropeptide Y neurons drive hedonic ingestive behaviour independent of energy homeostasis

**DOI:** 10.1038/s41366-026-02060-z

**Published:** 2026-04-03

**Authors:** Neda Rafiei, Caitlin S. Mitchell, Philip Jean-Richard-dit-Bressel, Caitlin R. Tedesco, Natasha N. Kumar, Gavan P. McNally, Herbert Herzog, Denovan P. Begg

**Affiliations:** 1 School of Psychology, University of New South Wales, Callaghan, NSW Australia; 2 School of Pharmacology, University of Sydney, Kensington, NSW Australia; 3 School of Biomedical Sciences and Pharmacy, University of Newcastle, Callaghan, NSW Australia; 4 School of Biomedical Sciences, University of New South Wales, Kensington, NSW Australia; 5 St. Vincent’s Centre for Applied Medical Research, Darlinghurst, Sydney Australia; 6 School of Clinical Medicine, Faculty of Medicine and Health, University of New South Wales, Kensington, NSW Australia

**Keywords:** Obesity, Feeding behaviour

## Abstract

**Background/objectives:**

Neuropeptide Y (NPY), a key orexigenic neurotransmitter, is widely expressed in the central nervous system, including in a distinct subpopulation of neurons within the central nucleus of the amygdala (CeA). While CeA NPY neurons contribute to energy regulation during chronic stress or high-fat diet exposure, the role of these neurons in modulating ingestive behaviour under standard conditions, particularly in response to caloric and non-caloric cues remains poorly understood.

**Subjects/methods:**

Using state-of-the-art chemogenetic techniques, we selectively activate NPY-expressing neurons in the CeA of NPY^Cre/+^ transgenic mice, enabling precise control of their activity in freely behaving animals.

**Results:**

Our experiments revealed that activation of these neurons significantly increased the consumption of both caloric and non-caloric palatable solutions, without affecting overall macronutrient preference. These findings indicate that CeA NPY neurons drive reward-related ingestive behaviour, promoting excess consumption beyond homoeostatic energy needs, regardless of the nutritional value of food. Importantly, this effect was observed independently of metabolic stress or dietary manipulation, suggesting that CeA NPY neurons engage a neural pathway that prioritizes food consumption based on reward value alone.

**Conclusion:**

This study provides novel insights into the neurobiological mechanisms underlying reward-driven consumption and identifies CeA NPY neurons as a key node in the neural circuitry mediating hedonic appetite. These findings have potential implications for understanding the pathophysiology of overeating and for developing targeted interventions for disorders characterized by dysregulated reward-based consumption.

## Introduction

NPY is expressed throughout the central nervous system and plays an essential role in regulating emotional and feeding behaviours. NPY is particularly concentrated in the hypothalamus, especially the arcuate nucleus (Arc), a region which plays an important role in regulating energy homeostasis [1, 2]. The orexigenic (appetite-stimulating) effects of hypothalamic NPY neurons in the Arc, including food consumption, motivation to eat, and food-seeking, have been the focus of much previous work [2–5]. However, NPY neurons can also be found outside the hypothalamus in other brain regions that are also known to modulate energy homeostasis, such as the central nucleus of amygdala (CeA).

The CeA has been best known for its function in regulating emotional (e.g., fear, anxiety) and addictive behaviours [6–8]. However, recently the CeA has also be implicated in modulating feeding behaviour. Interestingly, distinct neuronal circuits in the CeA have opposing functions in the regulation of energy homeostasis. Activation of CeA neurons which express serotonin receptor 2a (Htr2a) promotes food intake through a positive valence signal; inhibiting these neurons conversely reduces food consumption [9]. In contrast, protein kinase-C delta (PKC-δ+) neurons, located in the lateral CeA, have an anorexigenic effect on appetite, and chemogenetic activation of these neurons results in decreased food consumption [10]. While prepronociceptin (Pnoc) expressing CeA neurons have been identified to play an essential role in the consumption of palatable diets. The inhibition or ablation of CeA Pnoc neurons has a negative effect on the consumption of palatable food, such as a high-fat diet (HFD), and consequently reduces diet-induced obesity [11]. In addition, the abundance of melanocortin 4 receptors (MC4R) in the CeA and the vital role of these receptors in modulating energy intake provides additional evidence for the importance of the CeA in the regulation of feeding [12].

Recent research has significantly advanced our understanding of how NPY regulates feeding behaviour, particularly through its role in synaptic plasticity within the extended amygdala. These studies support the emerging view that NPY-expressing neurons in regions such as the central amygdala (CeA) integrate emotional and metabolic signals to modulate feeding, especially under conditions of stress or altered energy states [13–15]. Complementary evidence indicates that activation of CeA NPY neurons in chronically stressed animals with access to highly palatable, high-fat diets results in increased food intake, reduced energy expenditure, and subsequent obesity, underscoring their critical role in stress-induced feeding behaviours [13].

Further elucidating the mechanisms of NPY action demonstrated that during periods of starvation, NPY signalling in the bed nucleus of the stria terminalis (BNST) induces lasting changes in synaptic strength. Specifically, NPY enhances excitatory input onto GABAergic neurons via activation of Y1 receptors, thereby promoting feeding behaviour while concurrently suppressing competing behaviours such as social interaction and threat avoidance. Notably, these synaptic modifications persist even after the reintroduction of food, suggesting that NPY drives a long-term behavioural shift that prioritizes food-seeking in response to energy deficits. This form of plasticity reflects a neural adaptation designed to enhance the motivational salience of food and ensure energy restoration [14].

Despite these advances, the functional role of CeA NPY neurons under baseline, non-stressful conditions remain less well characterized. Therefore, we here explore how activation of these neurons in free-feeding paradigms affects broader aspects of energy homeostasis. These include parameters such as total food intake across different diet types, macronutrient preference, water and non-caloric food consumption, locomotor activity, and measures of emotional valence. Collectively, these findings contribute to a more comprehensive understanding of how NPY signalling within the extended amygdala regulates feeding behaviour and energy balance across varying physiological and environmental contexts.

## Materials and methods

### Animals

All experimental and animal care procedures were approved by the Animal Care and Ethics Committee at the University of New South Wales. All methods were performed in accordance with the relevant guidelines and regulations. Male and female NPY^Cre/+^ mice on a C57BL/6 background were bred at Australian BioResources and delivered to UNSW prior to 12 weeks of age. Upon arrival at the UNSW, mice were group housed in standard cages at 22 ± 2 °C with 12-h light and dark cycles. Animals had *ad libitum* access to water and standard chow (Specialty Feeds; 60% carbohydrate, 19% protein, 4.6% fat, digestible energy 3.4 kcal/gr) during experiments, unless otherwise described. All animals were acclimated and handled for one-week prior to the start of the experiments. In this study, equal numbers of male and female subjects were used to assess potential gender differences in the effects of chemogenetic activation of CeA NPY neurons on ingestive behaviour. Data from both sexes were combined, as no significant sex differences were detected in the experiments where activation of NPY neurons in the central amygdala influenced ingestive behaviours (Supplementary Figs. S1–5).

## Procedures

### Stereotaxic microinjection of the viral vector

Stereotaxic surgery was performed to deliver 200 nl of either a viral vector containing excitatory designer receptors exclusively activated by designer drugs (DREADD) AAV9-hSyn-DIO-hM3Dq-mCherry (Addgene # 44361) or a control virus AAV9-hSyn-DIO-mCherry (Addgene # 50459) into the CeA bilaterally (coordinates relative to the bregma: anterior-posterior, –1.06 mm; lateral, ±2.6 mm; dorsal-ventral, –4.75 mm; (George Paxinos, 1997; Ip et al., 2019). Viruses were injected at a speed of 100 nL/min using a 0.2 µL Hamilton syringe (World Precision Instruments Inc.). The needle was gradually withdrawn 4–5 min after the viral infusion. The wound was then cleaned and sutured using Prolene Suture (Size 5.0–6.0, Ethicon), and the animal was returned to its standard cage for recovery. Mice were given three weeks to recover and allow for viral expression before starting any experiments.

### Measuring food and liquid intake in the BioDAQ system

Three weeks after stereotaxic surgery, mice were individually housed in BioDAQ monitoring cages and provided with standard rodent chow. The BioDAQ system (Research Diets, NJ, USA) was used to continuously monitor ingestive behaviour, including metrics such as feeding and drinking bout number, bout size, duration, and post-bout intervals. The bouts were defined using two parameters: the inter-bout interval (IBI) and minimum food intake. In this study, an IBI of 300 s was used, meaning consummatory events separated by at least 5 min were considered distinct bouts. A minimum intake threshold of 0.02 grams was set to qualify as a bout.

Mice were given a one-week acclimation period in the BioDAQ cages before experimental testing. All experiments were conducted during the early light cycle in well-fed animals. Clozapine N-oxide (CNO; 1 mg/kg; Sigma-Aldrich, St. Louis, MO) or sterile saline (5 ml/kg) was administered intraperitoneally immediately before recording ingestive behaviour. A counterbalanced design was used, with a two-day washout period between CNO and saline treatments to ensure complete drug clearance.

Prior to macronutrient preference testing, mice were exposed ad libitum to 4% intralipid and 10% sucrose solutions for three consecutive days (6 h/day) in the BioDAQ cages. The 4% intralipid solution was prepared by diluting a 20% stock solution (Baxter Healthcare Corporation, Deerfield, IL), which contained 20% soybean oil, 1.2% egg yolk phospholipids, 2.25% glycerine, and water, with a caloric density of 2.0 kcal/ml. The 10% sucrose solution was prepared by dissolving sucrose in tap water, providing a caloric density of 0.4 kcal/ml. Saccharin solution was made up with 0.1 g of saccharin (Sigma-Aldrich, St. Louis, MO, 2,3-Dihydroxy-1, 2-benzisothiazol-3-one-1, 1-dioxide, 2-Sulfobenzoic acid imide, o-Benzoic sulfimide) dissolved in 100 milliliters of tap water.

For measuring water intake, we exposed the animals to the BioDAQ water bottle for 2–3 days, 6 h each day, before test days. We measured water intake during access to and the absence of food on two different experiment days. On the experiment day, we replaced the regular water bottle with a BioDAQ liquid hopper to allow us to monitor the drinking activity. All ingestive behaviour data were collected by BioDAQ and assessed cumulatively at 1-, 2-, 4-, and 6-h following drug administration, using BioDAQ Data Viewer software (Research Diets, New Brunswick, NJ).

Experimenters were blinded to treatment conditions during behavioural testing and data analysis whenever possible. In cases where blinding was not feasible, all analyses were conducted following standardized protocols to minimize potential bias.

### Locomotor activity

Mice were allowed to explore an open field (30 × 30 cm) for 30 min on three consecutive habituation days before test. On the test day, animals were injected with CNO or saline when food was removed from the home cage. Thirty minutes after injection, animals were placed in the open field with fresh bedding. A video camera installed above the open field recorded the animals’ activity. Locomotor activity data were analysed based on the distance from the centre of open field and each animal’s velocity using EthoVision software (X13).

### Conditioned place preference

The training and test sessions were performed in two-compartment place-preference chambers (29.8 cm width × 20.9 cm height × 20.3 cm length: Med Associates Inc. St Albans, VT, US). Each side of the chambers had a different floor style: a black grid rod-style floor in one compartment and a white mesh floor on the other side.

Mice were placed in chambers and were able to freely move between both compartments prior to conditioning. Animals’ positions were recorded, and the time spent in each chamber was assessed. If mice displayed any initial side preference, the most preferred compartment for each animal was assigned to saline injection and the other compartment to CNO injection. The conditioned place preference (CPP) protocol was sourced and modified from Alhadeff and colleagues [16, 17]. CPP training was conducted for 30 min each day on 10 consecutive days. During training, animals had access to only one of the compartments, counterbalanced between days where they first received CNO (A) and saline (B) injections on an ABBA schedule. The ABBA schedule was used to counterbalance the order of drug (CNO) and (saline) administration across training days. This design helps to minimize potential order or carryover effects that might arise if all animals received the same treatment sequence. By alternating the treatment days in an ABBA pattern, we ensured that any effects related to repeated exposure, habituation, or day-specific variability were evenly distributed between the CNO and saline sessions. Following training days, a test day was conducted during which animals were placed in the chamber with free access to both compartments. Animal movements and positions were tracked in the chamber and analysed using EthoVision software (X13).

### Perfusion

At the conclusion of the behavioural experiments, animals received an intraperitoneal injection of CNO (1 mg/kg). Two hours later, they were euthanized via a lethal injection of pentobarbital (120 mg/kg, i.p.; Sigma-Aldrich, St. Louis, MO) and transcardially perfused with 1× phosphate-buffered saline (PBS), followed by 4% paraformaldehyde solution (PFA/0.1 M PB). The brains were then extracted, post-fixed in 4% PFA solution for 2 h, and transferred to a 30% sucrose solution in PBS for at least 48 h. Subsequently, brains were sectioned at a thickness of 40 µm using a cryostat microtome at −18 °C, and the sections were stored in 0.1% sodium azide in PBS at 4 °C.

### Immunohistochemistry

Brain sections were rinsed three times with PBS and then incubated for 2 h in a blocking solution containing 10% normal goat serum (NGS, Sigma-Aldrich, St. Louis, MO) and 0.5% Triton X-100 in PBS. Following the blocking step, the sections were incubated for 24–48 h at 4 °C with a primary antibody, rabbit anti-c-Fos (1:500, Abcam), in a solution of 2% NGS and 0.2% Triton X-100 in PBS. After primary antibody incubation, the sections were washed three times with PBS and then incubated overnight at 4 °C with a secondary antibody, goat anti-rabbit (1:200, Alexa Fluor® 488, Abcam), prepared in 2% NGS and 0.2% Triton X-100 in PBS. The sections were then rinsed three additional times in PBS before being mounted onto glass microscope slides and cover slipped. c-Fos expression in the CeA was visualized using an Olympus FV1200 confocal microscope (Olympus, Tokyo, Japan).

### Sample size

The sample size for each experiment was determined based on previous studies in the field and power calculations to ensure sufficient statistical sensitivity. Power analysis was conducted using an expected effect size (Cohen’s d) derived from pilot data or published literature, with an alpha level set at 0.05 and desired power of 0.8 (80%). This approach ensures that the study is adequately powered to detect meaningful differences between experimental groups while minimizing the risk of Type II errors.

### Statistical design

Two-way repeated-measures ANOVA was used for all ingestive-behaviour experiments that included both a within-subject factor (drug: CNO vs saline) and a between-subject factor (viral group: hM3Dq vs mCherry), including analyses of standard chow intake, high-fat diet intake, macronutrient (intralipid vs sucrose) consumption, and caloric versus non-caloric (sucrose vs saccharin) solution intake. When significant main or interaction effects were detected, appropriate post-hoc multiple-comparison tests were applied. One-way ANOVA was used for analyses involving a single factor with more than two levels, such as locomotor activity and conditioned place preference. Two-tailed unpaired *t*-tests were used for between-group comparisons of single behavioural measures (e.g., feeding bout frequency and meal duration and gender). Variance was inspected across groups, and no substantial differences were observed.

## Results

### Chemogenetic activation of NPY neurons in the CeA increases standard food consumption

**Fig. 1:**
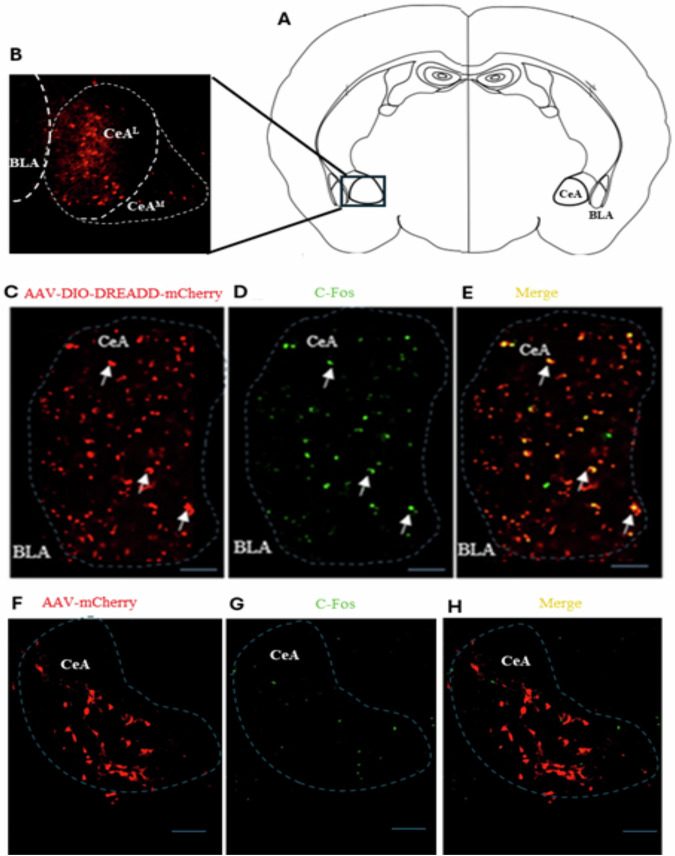
Stereotaxic microinjections targeting the central amygdala (CeA) in NPY-Cre mice. **A** Bilateral stereotaxic injections were performed in the CeA (coordinates relative to bregma: anterior–posterior, –1.06 mm; medial–lateral, ±2.6 mm; dorsal–ventral, –4.75 mm). **B** Representative photomicrograph showing hM3Dq-mCherry expression in the CeA. **C**, **F** Verification of AAV-hM3Dq and AAV-mCherry expression sites in NPY-Cre/+ animals. **D** Activation of NPY neurons expressing hM3Dq resulted in increased c-Fos protein expression in the CeA (arrows), whereas animals injected with AAV-mCherry did not show c-Fos activation (**G**). **E**, **H** Merged images demonstrate co-localization of hM3Dq-mCherry and c-Fos in the CeA of AAV-hM3Dq–injected animals (**E**), but not in AAV-mCherry controls (**H**). Scale bars: B = 50 µm; C-H 30 µm. CeA central nucleus of the amygdala, CeAL lateral division of the CeA, CeAM medial division of the CeA, BLA basolateral amygdala.

To examine the effect of activating CeA NPY neurons on standard chow consumption, two groups of NPY^Cre/+^ mice were injected with either a Cre-dependent excitatory DREADD virus (AAV-hM3Dq-mCherry) (*n* = 11) or a control virus (AAV-mCherry) (*n* = 5) into the CeA. The presence of the Cre-dependent AAV vector specifically in the CeA was confirmed by fluorescence microscopy of coronal brain sections of these mice (Fig. 1A, B). Animals were excluded from analysis if viral expression was not confined to the target brain region. Specifically, after histological verification, animals in which the viral expression was absent, off-target, or insufficiently expressed in the intended area were not included in the final dataset. The viral construct was localized exclusively in the CeA and did not spread to neighbouring regions, such as the basolateral amygdala (BLA). Our study did not include detailed spatial mapping of CeA NPY neurons, however the NPY-expressing neurons examined here appear to be predominantly localized within the lateral regions of the CeA (Fig. 1B).

**Fig. 2:**
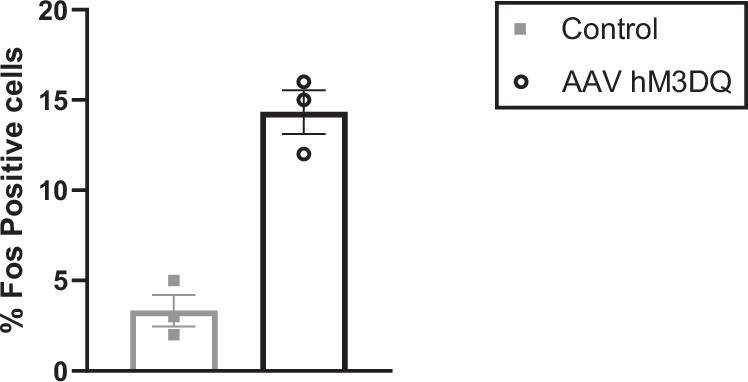
Animals expressing hM3Dq showed a significant increase in Fos colocalization compared with control animals injected with AAV-mCherry. Data are means ± SEM. *p < 0.05.

To assess the effects of chemogenetic activation of NPY neurons in the CeA on Fos expression, all animals received a CNO injection two hours prior to perfusion. Activation of NPY neurons in the CeA via CNO administration resulted in robust Fos expression in the CeA of NPY^Cre/+^ AAV hM3Dq animals (Fig. 1C–E) compared with control animals injected with AAV-mCherry (Fig. 1F–H). Additionally, there was notable colocalization of Fos protein expression with hM3Dq-transduced animals (*n* = 3) compared with animals in the control group injected with AAV-mCherry (*n* = 3) (*t* (2) = 5.50, *p* = 0.031) (Fig. 2).

**Fig. 3:**
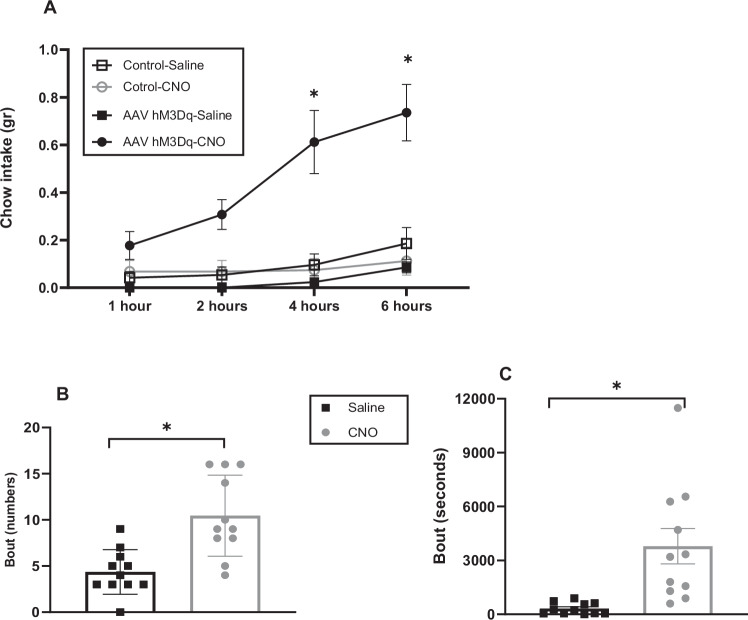
Activation of CeA NPY neurons promotes food consumption. **A** Consumption of standard chow increased significantly in NPYCre/+ AAV hM3Dq animals following CNO treatment but remained unchanged following saline treatment and control group. **B** Greater food consumption in NPYCre/+ AAV hM3Dq animals following CNO treatment compared with saline treatment was driven by increases in both **C** bout number and **D** meal duration. Data are means ± SEM. *p < 0.05.

Importantly, activation of CeA NPY neurons via DREADD stimulation following CNO administration resulted in a significant increase in food consumption compared to saline-treated animals and control group mice injected with AAV-mCherry (*F* (3, 112) = 37.34, *p* < 0.0001 (Fig. 3A). The increased food consumption in NPY^Cre/+^ AAV hM3Dq mice was attributable to a significant increase in bout frequency (*t* (10) = 4.18, *p* = 0.001) (Fig. 3B) and meal duration (*t* (10) = 3.6, *p* = 0.004) (Fig. 3C) following CNO treatment compared to saline.

### Chemogenetic activation of NPY neurons in the central amygdala increases the consumption of high-caloric palatable diet

**Fig. 4:**
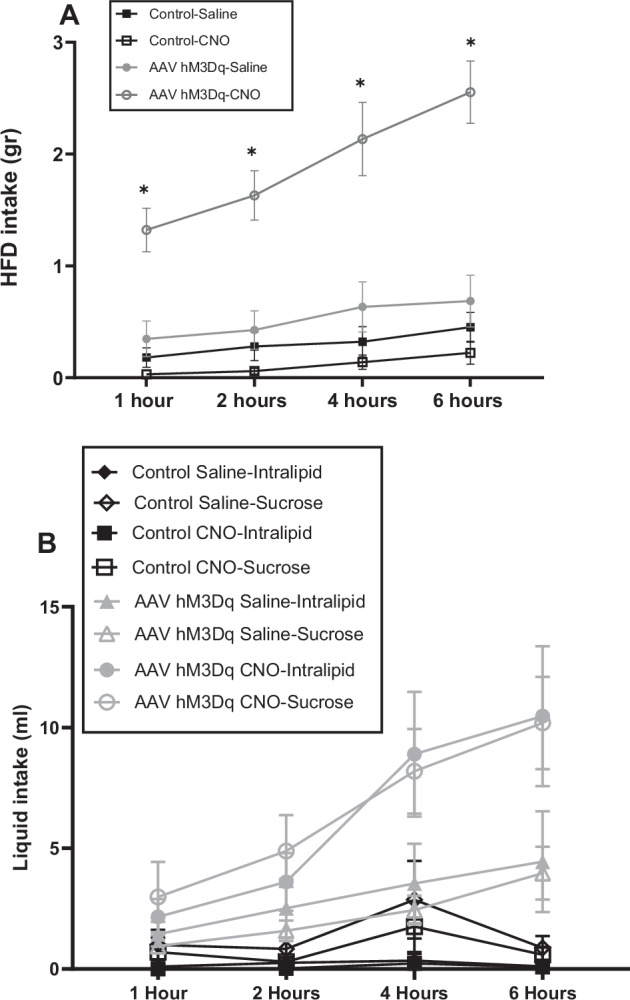
Chemogenetic activation of NPY neurons in the central amygdala increases consumption of a high-caloric palatable diet without affecting macronutrient preference. **A** Activation of NPY neurons via CNO administration in NPYCre/+ AAV-hM3Dq animals increased consumption of a high-caloric palatable diet relative to saline controls. **B** Activation of NPY neurons via CNO administration in NPYCre/+ AAV-hM3Dq animals had no effect on macronutrient preference between intralipid and sucrose intake. Data are presented as means ± SEM. *p < 0.05.

To examine the effects of stimulating CeA NPY neurons on the consumption of a palatable diet, all mice fed a high-fat diet for 3 days after which they were treated with IP injection of CNO or saline. As expected, NPY^Cre/+^ AAV hM3Dq mice ate significantly more of the HFD following CNO treatment (*F* (3, 112) = 53.92, *p* < 0.0001), compared with saline treatment and control group mice injected with AAV-mCherry (Fig. 4A),

### Chemogenetic activation of NPY neurons in the central amygdala does not affect macronutrient preference

The previous experiments showed that activating CeA NPY neurons increases food intake during access to standard or palatable diets. However, it remained unclear whether stimulating these NPY neurons in the CeA would influence preference between different macronutrients. To explore this, all mice were exposed to a 4% intralipid (fat) and 10% sucrose (carbohydrate) solutions, with each solution calorie-matched to control for any potential confounding factors such as form or caloric density.

Chemogenetic activation of CeA NPY neurons via CNO administration resulted in increased total consumption of both intralipid and sucrose solutions (*F* (7, 224) = 6.96, *p* < 0.0001) compared to saline treatment and control group mice injected with AAV-mCherry (Fig. 4B). However, despite the increase in overall food intake, there was no significant difference in the preference for intralipid versus sucrose, as both were consumed at similar levels following CNO treatment (*F* (1, 80) = 0.301, *p* = 0.58) (Fig. 4). These results suggest that CeA NPY neurons do not affect macronutrient preference.

### Chemogenetic activation of NPY neurons in the central amygdala only increases postprandial water consumption

**Fig. 5:**
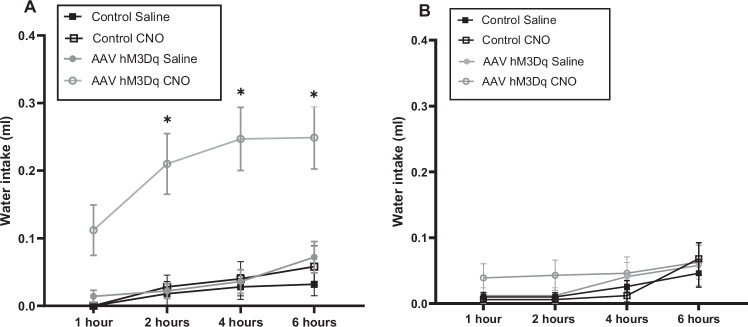
Chemogenetic activation of NPY neurons in the central amygdala increases postprandial water consumption. **A** Activating CeA NPY neurons via CNO administration increased water intake in the presence of food in NPYCre/+ AAV hM3Dq animals. **B** There was no significant difference in water intake between CNO and saline-treated test days in NPYCre/+ AAV hM3Dq animals when food was unavailable. Data are means ± SEM.

To examine the impact of stimulating CeA NPY neurons on water consumption, we measured water intake in all mice under two conditions: with food available and without food. In the presence of food, CNO treatment led to a significant increase in water consumption relative to saline treatment and control group (*F* (3, 104) = 3.255, *p* < 0.0001) (Fig. 5A). However, when food was unavailable, this effect was no longer observed. Water consumption did not significantly differ between CNO and saline treatments or control group in the absence of food (*F* (3, 104) = 1.17, *p* = 0.32) (Fig. 5B). This indicates the observed increase in water intake following CNO treatment is tied to the availability of food, rather than being a direct effect of CeA NPY neuron activation on water consumption.

### Chemogenetic activation of CeA NPY neurons increases non-caloric palatable liquid consumption

The findings from the previously described experiments indicated that activating CeA NPY neurons enhances the consumption of a palatable diet but does not appear to have a direct effect on water intake. These results raised the question of whether stimulation of these neurons could influence the consumption of a non-caloric sweetened solution, such as saccharin. To address this, we exposed all mice to a 0.1% saccharin solution in the BioDAQ cages, while administering IP injections of either CNO or saline on separate test days.

**Fig. 6:**
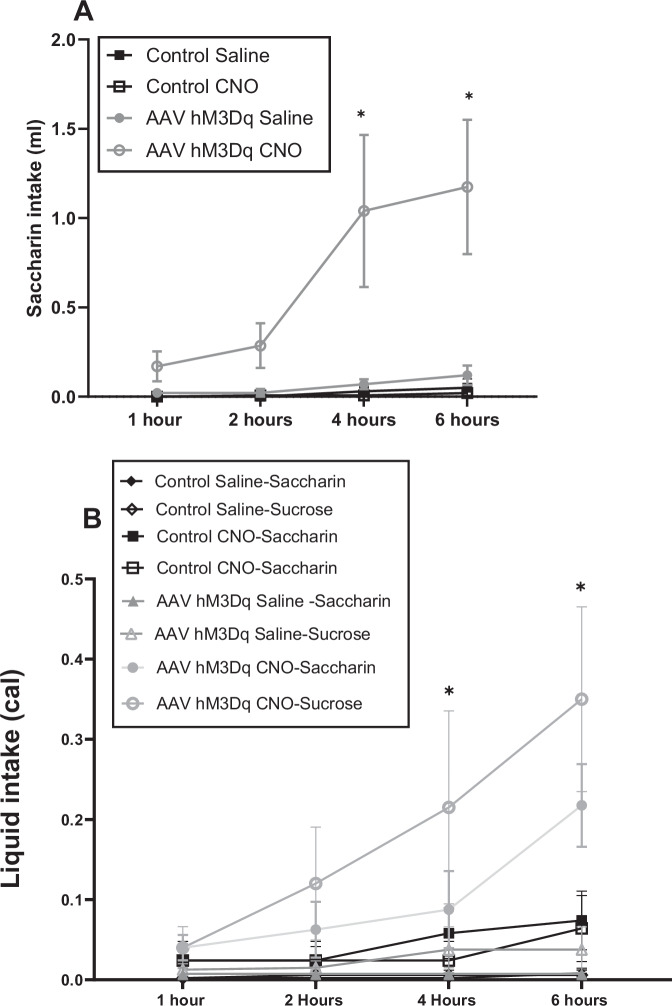
Chemogenetic activation of NPY neurons in the central amygdala increases consumption of both caloric and non-caloric solutions. **A** Activation of NPY neurons via CNO administration in NPYCre/+ AAV hM3Dq animals increased their intake of saccharin relative to saline control. **B** Activation of NPY neurons via CNO administration in NPYCre/+ AAV hM3Dq animals increased both sucrose and saccharin intake relative to a saline injection. Data are means ± SEM. *p < 0.05.

Remarkably, NPY^Cre/+^ AAV hM3Dq animals showed a significant increase in saccharin consumption following CNO administration compared to saline and control group (*F* (3, 48) = 12.61, *p* < 0.0001) (Fig. 6A). This suggests that activation of CeA NPY neurons not only enhances the intake of palatable foods but also promotes the consumption of a non-caloric solution.

### Chemogenetic activation of CeA NPY neurons increases consumption of both caloric and non-caloric palatable liquids

Our next objective was to determine whether activating CeA NPY neurons induces a preference between caloric (10% sucrose) and non-caloric (0.1% saccharin) sweetened solutions. To test this, we exposed all animals to both sucrose and saccharin solutions, administering IP injections of CNO and saline on separate test days.

The results revealed a significant increase in both sucrose and saccharin consumption in NPY^Cre/+^ AAV hM3Dq animals 6 h after CNO injection (*F* (7, 112) = 11.28, *p* < 0.0001) (Fig. 6B) compared to saline treatment and control group. These findings suggest that while activating CeA NPY neurons enhances the intake of both caloric and non-caloric sweetened solutions, it does not drive a preference for one over the other.

### Chemogenetic activation of NPY neurons in the central amygdala has no effect on locomotor activity

The previous experiments demonstrated that stimulating CeA NPY neurons increases energy intake, even in a fed state. However, it remained unclear whether activating these neurons influences locomotor activity. To address this, we measured the movement and velocity of NPY^Cre/+^ AAV hM3Dq animals (*n* = 10) and control animals injected with AAV-mCherry (*n* = 5) on CNO- and saline-treated test days.

**Fig. 7:**
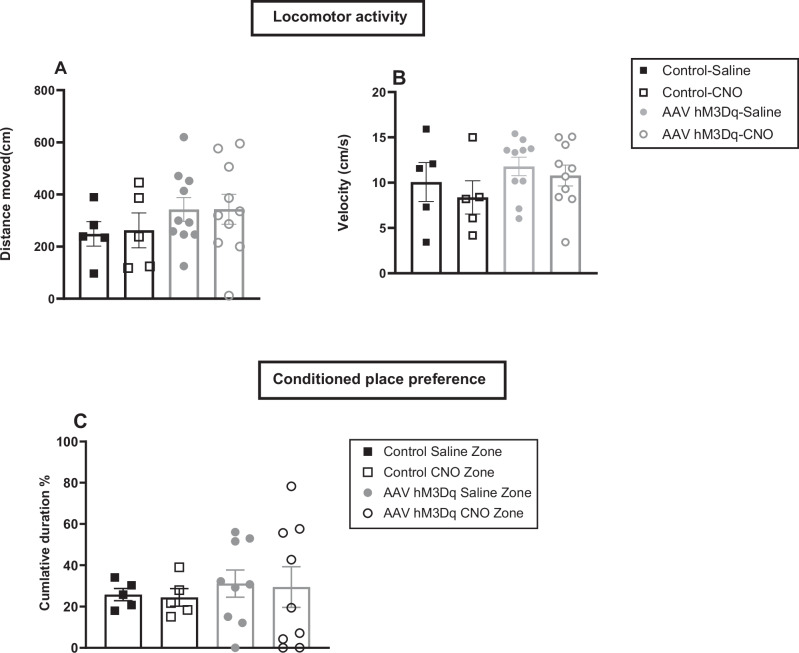
Chemogenetic activation of CeA NPY neurons does not affect locomotor activity and conditioned place preference or aversion. **A** NPYCre/+ AAV hM3Dq animals displayed no significant difference in distance moved between CNO and saline test days. **B** NPYCre/+ AAV hM3Dq animals displayed no significant difference in movement velocity between CNO and saline days. **C** There was no significant difference in the percentage of time that NPYCre/+ AAV hM3Dq animals spent in the CNO- and saline-paired compartments. Data are means ± SEM. *p < 0.05.

Our results showed no significant difference in the distance moved between CNO-treated and saline-treated test days in NPY^Cre/+^ AAV hM3Dq animals and control group (*F* (3, 26) = 0.72, *p* = 0.54) (Fig. 7A). Similarly, movement velocity did not differ between the CNO and saline-treated NPY^Cre/+^ AAV hM3Dq and control group (*F* (3, 26) = 0.83, *p* = 0.48) (Fig. 7B).

### Chemogenetic activation of CeA NPY neurons does not produce conditioned place preference (CPP) or conditioned place aversion (CPA)

We performed place-preference conditioning to investigate whether animals exhibit a conditioned place preference to the side of the chamber previously paired with CNO injection (i.e. paired with chemogenetic activation of CeA NPY neurons). Animals were trained in two-compartment chambers for ten days, during which they learned to associate each compartment with either CNO or saline injections. Our analysis revealed no significant difference in the time spent in the CNO- or saline-paired compartments in NPY^Cre/+^ AAV hM3Dq animals and control group (*n* = 9) (*F* (3, 24) = 0.14, *p* = 0.93) (Fig. 7C)

## Discussion

This study elucidates the role of NPY-expressing neurons in the central nucleus of the amygdala (CeA) in regulating ingestive behaviour and energy balance. Using chemogenetic approaches, we demonstrated that activation of CeA NPY neurons significantly increases consumption of both standard chow and a palatable high-fat diet in fed mice. These findings align with previous studies implicating the CeA in driving food intake [11, 13], particularly under conditions of chronic stress [13], and highlight the critical role of CeA NPY neurons in promoting hyperphagia, potentially contributing to obesity.

We further investigated whether CeA NPY neurons influence macronutrient intake and preference. Activation increased consumption of both fat and carbohydrate solutions without altering macronutrient preference. This contrasts with earlier reports showing that NPY administration in the amygdala enhances fat preference [18] and that amygdala lesions shift macronutrient preference in rats [19]. Methodological differences, such as our use of calorie-matched liquid diets to minimize confounds, may explain these discrepancies. The lack of macronutrient preference suggests that CeA NPY neurons broadly regulate palatability-driven consumption rather than selectively modulating macronutrient selection, distinguishing them from other CeA subpopulations [20, 21].

To determine whether CeA NPY neurons respond to caloric value or palatability, we assessed intake of caloric (sucrose) and non-caloric (saccharin) solutions. Activation increased consumption of both, indicating that these neurons drive ingestive behaviour primarily in response to hedonic properties rather than caloric content. This positions CeA NPY neurons as key regulators of reward-driven consumption independent of energy homeostasis.

We also examined the effects of CeA NPY neuron activation on water consumption. While activation increased water intake, this effect was contingent on food availability, suggesting it is a secondary consequence of increased food intake rather than a direct effect. This contrasts with other CeA populations, such as PKC-δ+ neurons, which inhibit water intake even under water-deprived conditions [10], highlighting the functional diversity of CeA circuits.

Notably, CeA NPY neuron activation did not alter locomotor activity, consistent with prior findings [22]. However, under chronic stress and high-fat diet exposure, these neurons reduce physical activity [9, 13], suggesting context-dependent behavioural effects.

Interestingly, activation did not induce conditioned place preference or aversion, indicating that CeA NPY neurons do not inherently produce a positive or negative valence state. This contrasts with other CeA populations, such as Htr2a-expressing neurons, which are inherently reinforcing [9]. The absence of valence changes in our study may reflect the food-dependent nature of CeA NPY neuron function, as our conditioned place preference test was conducted without food.

In summary, our findings demonstrate that activation of CeA NPY neurons is sufficient to promote consumption based on palatability, not caloric value, as evidenced by increased intake of both sucrose and saccharin. This suggests a primary role for this population in mediating the hedonic drive to feed. A key remaining question, however, is whether this function operates independently of energy homeostasis. To determine if CeA NPY neuron activity is necessary for feeding or is modulated by metabolic state, future work should employ inhibitory techniques (e.g., DREADDs) in subjects with varying energy needs (e.g., fed vs. fasted). This work advances our understanding of the neural circuits underlying food intake and identifies potential therapeutic targets for obesity and eating disorders.

## Supplementary information

Supplementary results

## Data Availability

The datasets generated and analysed during the current study are available from the corresponding author on reasonable request.
