# Supplementary material for: Central amygdala neuropeptide Y neurons drive hedonic ingestive behaviour independent of energy homeostasis
Source: Int J Obes (Lond). 2026 Apr 3;50(6):1267–75. doi: 10.1038/s41366-026-02060-z (PMC13287006; doi:10.1038/s41366-026-02060-z)
Supplement: Supplementary file 1 — Supplementary results [file 41366_2026_2060_MOESM1_ESM.docx]

**Supplementary data**

**CeA NPY stimulation effects on food consumption were independent of gender**

To explore potential gender differences in the function of NPY neurons on food consumption, we compared chow intake between male (n=6) and female (n=5) animals during Experiment 1. No significant difference in food intake was observed between the genders (t (9) = 0.172, p = 0.86) (Figure S1), indicating that gender does not affect the role of CeA NPY neurons in regulating energy intake.

Figure S1: Comparison of food intake between male and female mice. No significant difference in food consumption was observed between male and female CNO-treated NPY Cre/+ AAV hM3Dq mice. Data are means ± SEM.

**CeA NPY stimulation effects on HFD consumption were independent of gender**

To assess whether sex influences the role of NPY neurons in palatable diet, we compared high fat diet (HFD) consumption between male (n = 6) and female (n = 5) mice. The analysis revealed no significant difference in HFD intake between males and females (t (9) = 1.45, p = 0.18; Figure S2), suggesting that CeA NPY neuron activity affects energy intake similarly in both genders.

Figure S2: Comparison of high fat diet intake between male and female mice. No significant difference in HFD consumption was observed between male and female CNO-treated NPY Cre/+ AAV hM3Dq mice. Data are means ± SEM.

**CeA NPY stimulation effects on macronutrient preference were independent of gender**

To examine potential gender differences in macronutrient preference, we compared intake of intralipid and sucrose solutions between male (n = 6) and female (n = 5) mice. Statistical analysis showed no significant differences between sexes in the consumption of either intralipid or sucrose (F (1, 18) = 0.001, p = 0.96, Figure S3), suggesting that the influence of CeA NPY neuron activity on macronutrient preference does not differ between males and

female.

.

Figure S3: Comparison of macronutrients preference between male and female mice. No significant difference in macronutrient preference was observed between male and female CNO-treated NPY Cre/+ AAV hM3Dq mice. Data are means ± SEM.

**CeA NPY stimulation effects on water intake were independent of gender**

To examine potential sex differences in the role of NPY neurons in regulating water consumption, we compared water intake between male (n = 3) and female (n = 2) mice. The analysis revealed no significant difference in water intake between the sexes (t(8) = 0.73, p = 0.48; Figure S4), suggesting that CeA NPY neuron activity influences fluid consumption similarly in males and females.

**Figure S4.** Comparison of water intake between male and female mice.
No significant difference in water consumption was detected between male and female NPY-Cre/+ mice expressing AAV-hM3Dq treated with CNO. Data are presented as mean ± SEM.

**CeA NPY stimulation effects on saccharin intake were independent of gender**

To examine potential sex differences in the role of NPY neurons in non-caloric palatable diet we compared saccharin intake between male (n = 3) and female (n = 2) mice. The analysis revealed no significant difference in saccharin consumption between the sexes (t (3) = 0.035, p = 0.97; Figure S5), suggesting that CeA NPY neuron activity regulates sweet taste preference independently of sex.

Figure S5: Comparison of saccharin intake between male and female mice. No significant difference in saccharin consumption was observed between male and female CNO-treated NPY Cre/+ AAV hM3Dq mice. Data are means ± SEM.
